# DNA Damage Responses Are Induced by tRNA Anticodon Nucleases and Hygromycin B

**DOI:** 10.1371/journal.pone.0157611

**Published:** 2016-07-29

**Authors:** Sabrina Wemhoff, Roland Klassen, Anja Beetz, Friedhelm Meinhardt

**Affiliations:** 1 Institut für Molekulare Mikrobiologie und Biotechnologie, Westfälische Wilhelms-Universität Münster, Münster, Germany; 2 Institut für Biologie, Fachgebiet Mikrobiologie, Universität Kassel, Kassel, Germany; University of Leicester, UNITED KINGDOM

## Abstract

Previous studies revealed DNA damage to occur during the toxic action of PaT, a fungal anticodon ribonuclease (ACNase) targeting the translation machinery via tRNA cleavage. Here, we demonstrate that other translational stressors induce DNA damage-like responses in yeast as well: not only zymocin, another ACNase from the dairy yeast *Kluyveromyces lactis*, but also translational antibiotics, most pronouncedly hygromycin B (HygB). Specifically, DNA repair mechanisms BER (base excision repair), HR (homologous recombination) and PRR (post replication repair) provided protection, whereas NHEJ (non-homologous end-joining) aggravated toxicity of all translational inhibitors. Analysis of specific BER mutants disclosed a strong HygB, zymocin and PaT protective effect of the endonucleases acting on apurinic sites. In cells defective in AP endonucleases, inactivation of the DNA glycosylase Ung1 increased tolerance to ACNases and HygB. In addition, Mag1 specifically contributes to the repair of DNA lesions caused by HygB. Consistent with DNA damage provoked by translation inhibitors, mutation frequencies were elevated upon exposure to both fungal ACNases and HygB. Since polymerase ζ contributed to toxicity in all instances, error-prone lesion-bypass probably accounts for the mutagenic effects. The finding that differently acting inhibitors of protein biosynthesis induce alike cellular responses in DNA repair mutants is novel and suggests the dependency of genome stability on translational fidelity.

## Introduction

The aminoglycoside antibiotic hygromycin B (HygB) from *Streptomyces hygroscopicus* displays activities against pro- and eukaryotic organisms [1,2]; it possesses unique structural and functional properties [3, 4]. Unlike other aminoglycosides, HygB has a dual impact on mRNA translation. It primarily interferes with the ribosomal translocation process by hampering tRNA and mRNA movements on the ribosome [5–7] and—as for other aminoglycoside antibiotics—it disturbs aminoacyl-tRNA recognition by distorting the ribosomal decoding center (A-site). Thus, HygB not only inhibits ribosomal translocation but promotes codon misreading as well, even though to a lesser extent than other aminoglycoside antibiotics, such as neomycin, paromomycin or gentamycin [2,7–8].

Anticodon nucleases (ACNases) acting as killer toxins of the yeasts *Pichia acaciae* and *Kluyveromyces lactis* (PaT and zymocin, respectively) [9, 10] are encoded by virus like elements (VLEs) which persist in the cytoplasm of the respective host cells [11–13]. Specific immunity proteins ensure stable propagation of the extranuclear elements via autoselection [14–16]. Secreted heteromeric killer toxins bind to the target cell’s chitin via carrier subunits [17–20] which subsequently release their toxic cargo, i.e. the ACNases, into target cells where they cleave specifically tRNA^Gln^ (PaT) or tRNA^Glu^ (zymocin) respectively [9, 10].

PaT action not only disables translation at the step of tRNA supply but also interferes with genome integrity [10, 21–23]. Mutation rates are enhanced upon PaT exposure and the DNA damage checkpoint kinase Rad53 is activated, ultimately resulting in a cell cycle arrest in the S-phase, followed by programmed cell death [23, 24]. Genetic analysis of mutants defective in various DNA repair pathways, such as base excision repair pathway (BER), homologous recombination (HR), non-homologous end-joining (NHEJ) and post replication repair (PRR) revealed evidence for the accumulation of apurinic (AP) sites and the formation of replication fork stalling derived DNA double strand breaks (DSB) upon PaT exposure [21, 22]. While BER and HR are probably involved in repairing the toxic DNA lesions induced by the killer toxin, thereby promoting resistance, PRR represents an important alternative for handling stalled forks by preventing their collapse into DSBs [21]. Even though PaT and zymocin target different tRNA species, several DNA repair mutants responded rather uniformly to both toxins, suggesting that loss of DNA integrity might be a general effect of tRNA cleavage [10, 21]. Only rather recently, we reported that loss of DNA integrity seen upon PaT treatment is caused by a mechanism that involves the depletion of the highly and periodically S-phase specific ribonucleotide reductase (RNR) as a consequence of specific tRNA offence [25]. Reduced dNTP levels cause replication fork stalling and collapse into DSBs [10, 22]. Interestingly, RNR is not only affected by PaT, but also by another translational inhibitor paromomycin [26], which led us to surmise that translational stress may disturb the target cell’s DNA integrity in general. Here, we provide genetic evidence for a possible general and novel principle that links genome stability to translational fidelity.

## Materials and Methods

### Strains, growth conditions and transformation

Strains used in this study are listed in Table 1. Yeasts were grown in YPD (2% peptone, 1% yeast extract, 2% glucose) at 30°C. Transformation of *S*. *cerevisiae* CEN.PK2-1c was performed by the LiAc / SS carrier DNA / PEG method according to [27]. Mutants were selected on YPD with 200 μg ml^-1^ G418 or on YNB (0.67% YNB w/o AA, Carbohydrate & w/AS (Y2025; BIOMOL, Hamburg, Germany), 2% glucose) with 30 μg ml^-1^ L-leucine, 20 μg ml^-1^ L-histidine, 20 μg ml^-1^ L-methionine, 20 μg ml^-1^ L-tryptophan or 20 μg ml^-1^ uracil when required. Gene disruption cassettes were generated by PCR (primers -koF/-koR) with plasmids pUG6 (*kanMX*), pUG27 (*SpHIS5*) or pUG72 (*KlURA3*) (Tables 2 and 3) [28]. Gene deletions were verified by PCR with primers located outside of the target genes (-outF/-outR) and/or marker specific primers (-up/-down) (Table 2).

**Table 1:** Strains used in this study.

Strain	Genotype	Reference
*Kluyveromyces lactis* AWJ137	*MAT*a *leu2 trp1* [pGKL1^+^, pGKL2^+^]	[29]
*Pichia acaciae* NRRL Y-18665	wild type [pPac1-1^+^, pPac1-2^+^]	[11]
*Saccharomyces cerevisiae* CEN.PK2-1c	*MAT*a *leu2-3*,*112 ura3*-*52 trp1*-*289 his3Δ1 MAL2*-*8*^*c*^ *SUC2*	[30]
*S*. *cerevisiae* CEN.PK2-1c *apn1*	as CEN.PK2-1c, but *apn1*::*HIS3*	[33]
*S*. *cerevisiae* CEN.PK2-1c *apn2*	as CEN.PK2-1c, but *apn2*::*hisG*	[33]
*S*. *cerevisiae* CEN.PK2-1c *apn1 apn2*	as CEN.PK2-1c, but *apn1*::*HIS3 apn2*::*hisG*	[33]
*S*. *cerevisiae* CEN.PK2-1c *apn1 apn2 mag1*	as CEN.PK2-1c, but *apn1*::*HIS3 apn2*::*hisG mag1*::*KlURA3*	[21]
*S*. *cerevisiae* CEN.PK2-1c *apn1 apn2 ung1*	as CEN.PK2-1c, but *apn1*::*HIS3 apn2*::*hisG ung1*::*KlLEU2*	[21]
*S*. *cerevisiae* CEN.PK2-1c *apn1 rad1*	as CEN.PK2-1c, but *apn1*::*HIS3 rad1*::*kanMX4*	[21]
*S*. *cerevisiae* CEN.PK2-1c *apn1 rad1 ntg1 ntg2*	as CEN.PK2-1c, but *apn1*::*HIS3 apn2*::*hisG ntg1*::*KlURA3 ntg2*::*KlLEU2*	[21]
*S*. *cerevisiae* CEN.PK2-1c *apn1 apn2 rev3*	as CEN.PK2-1c, but *apn1*::*HIS3 apn2*::*hisG rev3*::*kanMX4*	This work
*S*. *cerevisiae* CEN.PK2-1c *apn1 apn2 rev3 rad30*	as CEN.PK2-1c, but *apn1*::*HIS3 apn2*:.*hisG rev3*::*kanMX4 rad30*::*KlURA3*	This work
*S*. *cerevisiae* CEN.PK2-1c *mag1*	as CEN.PK2-1c, but *mag1*::*hisG*	[34]
*S*. *cerevisiae* CEN.PK2-1c *ntg1*	as CEN.PK2-1c, but *ntg1*::*KlURA3*	[21]
*S*. *cerevisiae* CEN.PK2-1c *ntg2*	as CEN.PK2-1c, but *ntg2*::*KlLEU2*	[21]
*S*. *cerevisiae* CEN.PK2-1c *ntg1 ntg2*	as CEN.PK2-1c, but *ntg1*::*KlURA3 ntg2*::*KlLEU2*	[21]
*S*. *cerevisiae* CEN.PK2-1c *ogg1*	as CEN.PK2-1c, but *ogg1*::*KlURA3*	[21]
*S*. *cerevisiae* CEN.PK2-1c *rad1*	as CEN.PK2-1c, but *rad1*::*kanMX4*	[21]
*S*. *cerevisiae* CEN.PK2-1c *rad5*	as CEN.PK2-1c, but *rad5*::*KlURA3*	[33]
*S*. *cerevisiae* CEN.PK2-1c *rad6*	as CEN.PK2-1c, but *rad6*::*KlURA3*	[34]
*S*. *cerevisiae* CEN.PK2-1c *rad18*	as CEN.PK2-1c, but *rad18*::*HIS3MX6*	[33]
*S*. *cerevisiae* CEN.PK2-1c *rad30*	as CEN.PK2-1c, but *rad30*::*KlURA3*	This work
*S*. *cerevisiae* CEN.PK2-1c *rad51*	as CEN.PK2-1c, but *rad51*::*KlLEU2*	[33]
*S*. *cerevisiae* CEN.PK2-1c *rad52*	as CEN.PK2-1c, but *rad52*::*SpHIS5*	This work
*S*. *cerevisiae* CEN.PK2-1c *rad55*	as CEN.PK2-1c, but *rad52*::*KlLEU2*	[33]
*S*. *cerevisiae* CEN.PK2-1c *rad59*	as CEN.PK2-1c, but *rad55*::*KlURA3*	[22]
*S*. *cerevisiae* CEN.PK2-1c *rev3*	as CEN.PK2-1c, but *rev3*::*kanMX4*	[34]
*S*. *cerevisiae* CEN.PK2-1c *rev3 rad30*	as CEN.PK2-1c, but *rev3*::*kanMX4 rad30*::*KlURA3*	This work
*S*. *cerevisiae* CEN.PK2-1c *srs2*	as CEN.PK2-1c, but *srs2*::*KlURA3*	[21]
*S*. *cerevisiae* CEN.PK2-1c *ung1*	as CEN.PK2-1c, but *ung1*::*KlLEU2*	[21]
*S*. *cerevisiae* CEN.PK2-1c *yku80*	as CEN.PK2-1c, but *yku80*::*SpHIS5*	This work
*S*. *cerevisiae* GA-180	*MAT*a *ade2-1 trp1-1 his3-11*,*15 ura3-1 leu2*-*3*,*112 can1*-*100*	[31]
*S*. *cerevisiae* GA-1230	*MAT*a *rad53*-*11 ade2*-*1 trp1*-*1 his3*-*11*,*15 ura3*-*1 leu2-3*,*112 can1*-*100 bar1*::*hisG ssd1-d2*	[31]
*S*. *cerevisiae* KY117	*MAT*a *ura3*-*52 trp1*-*Δ1 lys2*-*801*^*am*^ *ade2*-*101 his3*-*Δ200*	[32]

**Table 2:** Primer used in this study.

Primer	Sequence (5’-3’)
RAD30-koF	GGAGTTGATTCAGCTTGGTTCCCCCAGTAAAGCATACGAGTCCTCCAGCTGAAGCTTCGTACGC
RAD30-koR	TGTTTTTGGAAGATGTAACTTGTTTCTTCTGAGGTGTGGCAGTATGCATAGGCCACTAGTGGATCTG
RAD30-outF	CCTGCCGATCATAGGATACC
RAD30-outR	GGCGCCCGTGAATCATTTAG
RAD52-koF	ATGGCGTTTTTAAGCTATTTTGCCACTGAGAATCAACAAATGCAACAGCTGAAGCTTCGTACGC
RAD52-koR	ATAATGATGCAAATTTTTTATTTGTTTCGGCCAGGAAGCGTTTCCGCATAGGCCACTAGTGGATCTG
RAD52-outF	TCTGCTCTTCCCGTTAGTG
RAD52-outR	TTTGTTTCGGCCAGGAAGC
REV3-koF	ATGTCGAGGGAGTCGAACGACACAATACAGAGCGATACGGTTAGACAGCTGAAGCTTCGTACGC
REV3-koR	ATTACCAATCATTTAGAGATATTAATGCTTCTTCCCTTTGAACAGGCATAGGCCACTAGTGGATCTG
REV3-outF	TCGCTCCTTTGTTCCATTCC
REV3-outR	CCACTCTTAGAGGATACG
YKU80-koF	TATCTCACACCATAATAATGTCAAGTGAGTCAACAACTTTCATCCAGCTGAAGCTTCGTACGC
YKU80-koR	AGATGGTCACGGGAATGTATGACGATCCAGACTGGTCATCTTCACGCATAGGCCACTAGTGGATCTG
YKU80-outF	CCGTCAGGGCATTTGTTGTC
YKU80-outR	CACCATAACGGTATGCCTTC
HIS5-up	GCCATGCGCGCGGCTAC
HIS5-down	GTAGCCGCGCGCATGGC
LEU2-up	GGCGTATAGACCCAATTCC
LEU2-down	GGAATTGGGTCTATACGCC
KanMX-up	GATGACGAGCGTAATGGCT
KanMX-down	AGCCATTACGCTCGTCATC
URA3-up	GACGCTGGCGTACTGGC
URA3-down	GCCAGTACGCCAGCGTC

**Table 3:** Plasmids used in this study.

Plasmid	Genotype	Reference
pUG27	*loxP-SpHIS5-loxP*, *Amp*^*R*^, *E*. *coli* ori	[28]
pUG72	*loxP-KlURA3-loxP*, *Amp*^*R*^, *E*. *coli* ori	[28]
pUG6	*loxP-kanMX-loxP*, *Amp*^*R*^, *E*. *coli* ori	[28]

### Killer toxin and DNA damage assay

Killer toxins were partially purified from supernatants of *Pichia acaciae* NRRL Y-18665 or *Kluyveromyces lactis* AWJ1347 stationary phase cultures by ultrafiltration using Vivaspin 20ml centrifugal devices with 100 kDa cutoff membranes (Sartorius Stedim Biotech GmbH, Göttingen, Germany). Killer assays were performed as previously described [22]. Microtiter assays were performed in 200μl YPD with increasing concentrations of PaT, zymocin or the ribosomal inhibitors neomycin, HygB, cycloheximide, geneticin or paromomycin. Inoculation was performed with 1μl preculture and incubated for 16 h at 30°C. The relative concentration factors (RCF) of PaT and zymocin were determined based on the concentration obtained by ultrafiltration. An RCF of 1 corresponds to the toxin concentration in non-concentrated supernatants of stationary phase cultures [35]. Relative growth was monitored photometrically at 620 nm (Multiscan FC, Thermo Fisher Scientific Oy, Vantaa, Finland) and refers to the OD_620_ value of strains incubated in toxin-free medium. The data are based on three biological replicates with two technical replicates for each value. For drop dilution assays, YPD plates were supplemented with different concentrations of PaT or HygB. A series of 10-fold dilutions of yeast cells, primarily adjusted to identical OD values, were dropped onto plates and incubated for 2–3 days at 30°C.

### Determination of mutation rates

For determining the mutation frequency at the *CAN1* locus following toxin exposure, the canavanine mutation assay was applied as previously described [23]. *S*. *cerevisiae* CEN.PK2-1c cells were incubated in YPD or in medium containing PaT (RCF 1), zymocin (RCF 1) or HygB (50 mM). After cultivation for 4 h at 30°C, cells were washed twice with sterile water. Aliquots of serial dilutions were subsequently plated on YPD medium to determine the number of viable cells, or on YNB lacking L-arginine but containing 5 μg ml^-1^ L-canavanine to determine the number of canavanine resistant cells. Following incubation for 2 days at 30°C, colony forming units were counted and the mutation frequency was expressed as the number of canavanine resistant cells per 10^6^ viable cells. The assay was repeated at least sevenfold.

### Determination of the budding index

Early log-phase cells of *S*. *cerevisiae* KY117 were synchronized in G1 using YPD supplemented with 5 μg ml^-1^ α-factor [24]. Release from G1-arrest was checked by monitoring the budding index [23]. Following incubation with the α-pheromone for 2 h at 30°C, cells were washed twice with sterile water and released to YPD only or YPD containing PaT (RCF 35) or HygB (100 mM). Over a period of 240 min, samples were taken at timely intervals and cells were immediately fixed in 70% ethanol for 2 h at room temperature. Following rehydration in phosphate buffered saline, a minimum of 300 cells per sample were used to determine the budding index that is expressed as the number of budded cells per total cell count. The assay was repeated threefold.

### Detection of histone H2A phosphorylation

*S*. *cerevisiae* KY117 cells were synchronized in G1 using 5 μg ml^-1^ α-pheromone (Sigma-Aldrich, Steinheim, Germany) and the release from G1-arrest was monitored by determining budding indices [23]. Samples were taken immediately at the G1-arrest. After washing twice with sterile water, cells were released to YPD only or to medium containing either HygB (0.1 mM), methyl methanesulfonate (MMS, 0.2%) or PaT (RCF 35). Samples were collected after 30 min (MMS) and after 5, 60, 120 and 180 min (YPD, HygB and PaT). Equal amounts of total protein fractions were separated in 15% polyacrylamide gels and blotted onto polyvinylidene difluoride (PVDF) membranes or stained with coomassie brilliant blue [21]. Detection of phosphorylated histone H2A at serine 129 was accomplished using an anti-histone H2A (phospho S129) antibody (ab15083; Abcam, Cambridge, UK).

## Results and Discussion

### DNA repair confers resistance to ribosomal inhibitors

**Fig 1:**
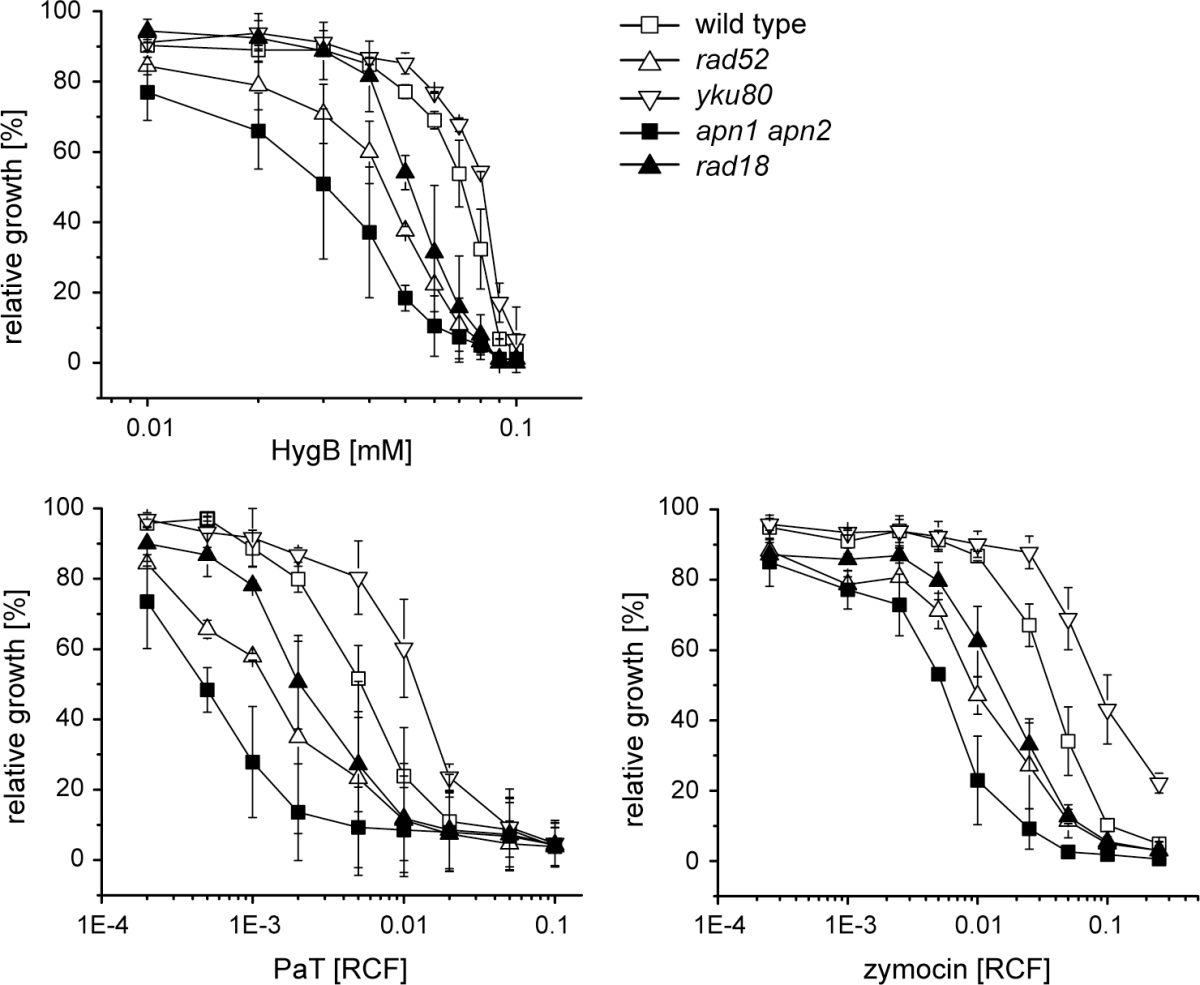
DNA repair mechanisms contribute to resistance against translational inhibitors. Microtiter assays were performed with *S*. *cerevisiae* strains deficient in homologous recombination (*rad52*), non-homologous end-joining (*yku80*), base excision repair (*apn1 apn2*) or post replication repair (*rad18*). Relative growth was determined photometrically at 620 nm and corresponds to the OD_620_ value of strains cultivated in toxin-free medium. Error bars are standard deviations of three biological replicates with two technical replicates each.

Specific tRNA offence (tRNA^Gln^) by ACNase action does not only impair translation but also affects genome integrity of target cells as DNA repair was found to significantly contribute to cell survival after exposure to the ACNases [10, 21, 22]. For checking effects of other translational inhibitors, DNA repair mutants defective in homologous recombination (HR, *rad52*), non-homologous end-joining (NHEJ, *yku80*), post replication repair (PRR, *rad18*), and base excision repair (BER, *apn1 apn2*) were analyzed with respect to their response to ribosomal inhibitors, among them HygB (Fig 1) but also paromomycin, neomycin, geneticin and cycloheximide (S1 Fig). Obtained sensitivity profiles were compared to both ACNase toxins (Fig 1). For phenotypic verification, the generated mutants were checked with respect to their reported UV, MMS and hydroxyurea (HU) sensitivity (S2 Fig).

Hypersensitivity of the mutant defective in Apn1 and Apn2 revealed a HygB protective effect of AP endonucleases involved in BER (Fig 1). Results obtained with both of the ACNase toxins agree with previous findings [10, 21]. The *rad18* mutant, defective in initiating PRR, displayed hypersensitivity to HygB. A similar outcome, i.e. hypersensitivity, was obtained with the *rad52* mutant defective in recombinational DSB repair as previously shown and confirmed here for the ACNases [10, 22]. Inactivation of Yku80 involved in NHEJ, a competing but error-prone DSB repair pathway that involves direct ligation of broken ends without relying on homologous templates, caused partial resistance to the yeast ACNase toxins (Fig 1). This effect was previously demonstrated to be specific to the loss of Yku80 but not NHEJ per se [10, 22]. Since Yku80 competes with Rad52 for binding DSB ends, its loss channels such ends towards the error-free HR pathway, thereby enhancing toxin tolerance. For HygB, at least a minor protective effect of the NHEJ-mutant (*yku80*) became evident (Fig 1).

DNA repair protected against other ribosomal inhibitors as well, even though to a lesser degree (S1 Fig). Geneticin, in particular, provoked a reaction of repair mutants quite similar, though not identical, to ACNases and HygB. Inactivation of Apn1/2, Rad18 and Rad52 increased geneticin toxicity, whereas deletion of *YKU80*, however, did not detectably alter sensitivity. Unlike geneticin, a great disparity was observed in the mutants’ responses to neomycin, paromomycin and cycloheximide (S1 Fig). Mutations in *APN1/2* and *YKU80* did not impact neo- and paromomycin toxicity, whereas Rad18 appeared to be dispensable for cycloheximide survival (S1 Fig). Accordingly, distinct DNA repair mechanisms affect resistance of the drugs used, though in a different manner. Thus, translational inhibitors probably exert effects on the genome stability, but cells cope differently with the concomitantly occurring DNA damage. As HygB triggered the most pronounced reaction, similar to those induced by the yeast ACNases PaT and zymocin, we focused on the hitherto unknown (and unanticipated) HygB-mediated impact on genome integrity.

### Mutagenesis induced by translational inhibitors

**Fig 2:**
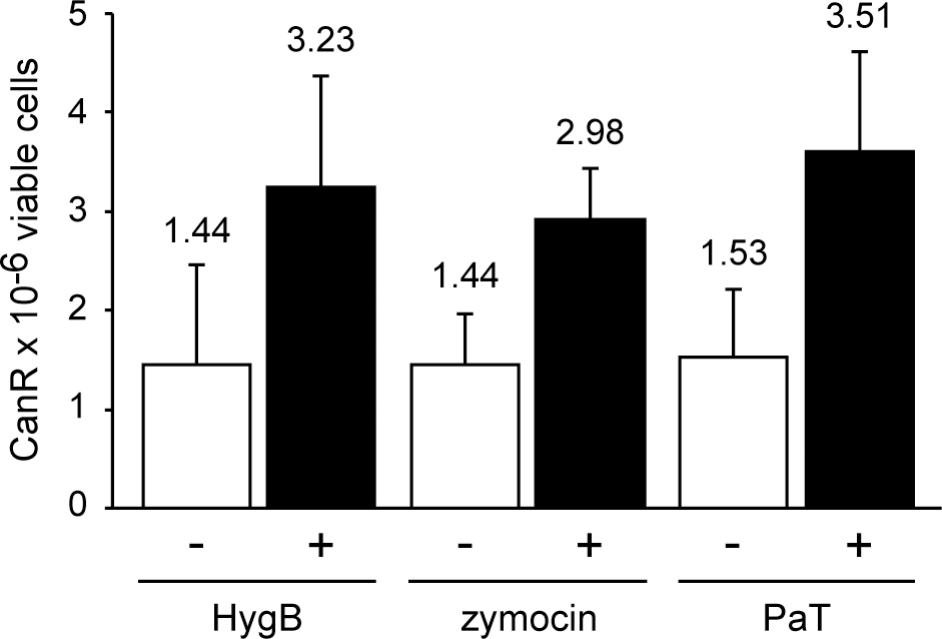
*CAN1* mutations induced by translational inhibitors. The mutation frequencies at the *CAN1* locus were monitored in *S*. *cerevisiae* cells exposed to hygromycin B (HygB, 50 mM), zymocin or PaT (RCF 1). A relative concentration factor of 1 (RCF 1) equals the toxin concentration in the supernatant of a stationary phase culture of *P*. *acaciae* or *K*. *lactis*. The mutation frequencies were determined as the number of canavanine resistant cells (CanR) per 10^6^ viable cells. Standard deviations are depicted as error bars.

As mutation frequencies increased upon PaT exposure [23], we checked whether zymocin and HygB promote mutagenesis as well (Fig 2). Random and toxin induced mutation rates at the *CAN1* locus of *S*. *cerevisiae* were read. Indeed, cells challenged with either HygB, zymocin or–as a positive control PaT—yielded a higher number of mutants than the negative (mock) control. Results obtained for both, HygB and zymocin, revealed not only mutagenic effects of a tRNA-endonuclease other than PaT, but also the differently acting translational inhibitor HygB is mutagenic, which agrees with DNA repair pathways being generally involved in conferring resistance to inhibitors of protein biosynthesis (Fig 1).

### Hygromycin B provokes a cell cycle arrest in the S-phase

**Fig 3:**
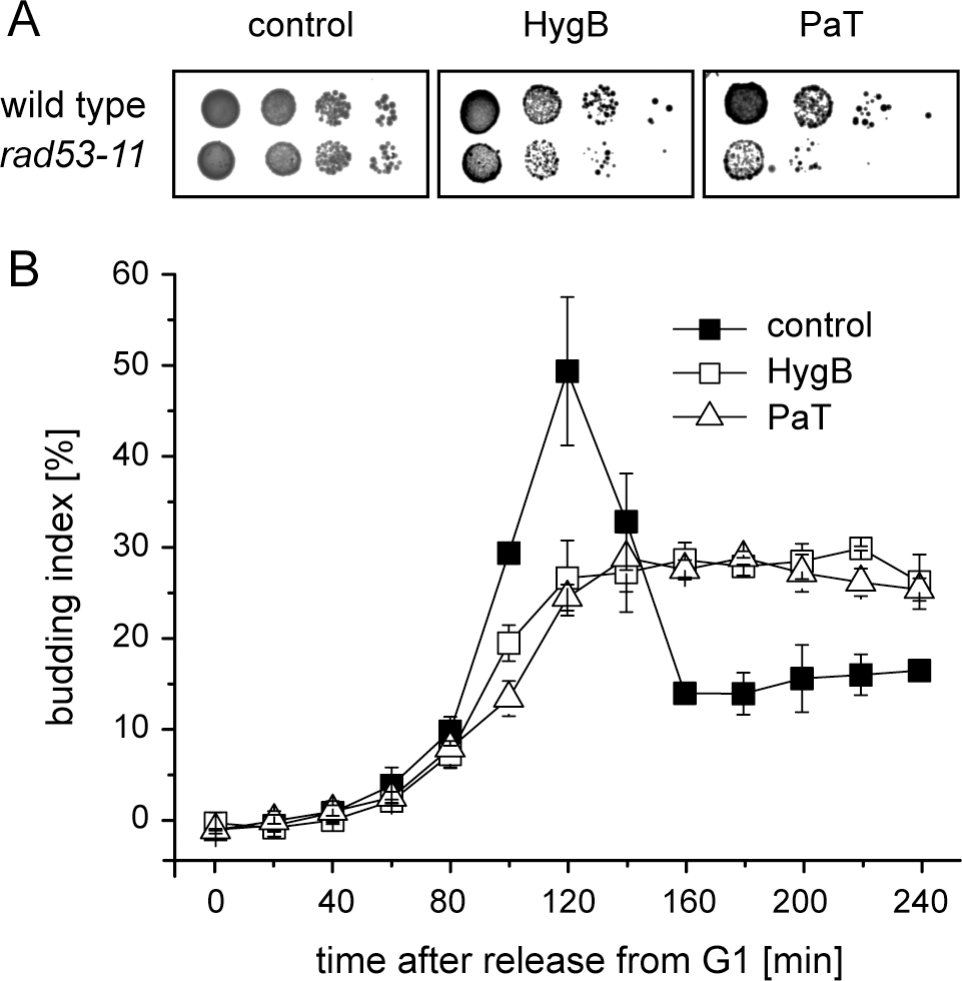
S-phase arrest induced by hygromycin B. (A) Relevance of the Rad53 checkpoint kinase for Hygromycin B action. *S*. *cerevisiae* GA-180 (wild type) and isogenic *S*. *cerevisiae* GA-1230 (*rad53-11*) cells were analyzed by drop dilution assays using YPD plates supplemented with PaT (RCF 0.1) or HygB (2 mM) B. (B) Budding kinetics of cells exposed to PaT or HygB. *S*. *cerevisiae* KY117 cells were synchronized in G1 with the α-factor and released to YPD (mock) or medium containing PaT (RCF 35) or HygB (0.1 mM). The relative budding index [%] was determined as the number of budded cells per total cell counts. Error bars indicate standard deviations of three biological replicates.

As a cellular response to DNA damage and replication stress the intra S-phase DNA damage checkpoint is activated. The respective key regulator is the effector-kinase Rad53, which—among others—regulates firing of late replication origins, stabilization of stalled replication forks, cell cycle progression, and transcription of genes instrumental in the DNA damage response [36–39]. Replication fork stalling by PaT involves an S-phase arrest by activating the Rad53 checkpoint kinase [22–24]. For checking the involvement of Rad53 in the cell’s reaction to HygB, its effect on growth of a *S*. *cerevisiae* mutant carrying the checkpoint deficient allele *rad53-11* was monitored in a drop dilution assay. The increased sensitivity of the mutant indicates that a functional Rad53 checkpoint kinase is indeed vital for HygB survival, as for the ACNase PaT (Fig 3A).

The budding indices of synchronized HygB treated cells were determined and compared to PaT and mock samples. Synchronization of cell division was performed by arresting the cells in G1 with the alpha pheromone. Subsequent release to cell cycle progression was done either in toxin-free (mock) or in medium containing HygB or PaT (Fig 3B). Cells immediately started budding, indicating their transition into the S-phase; the budding index increased from 0% to 50% (mock) and rapidly decreased upon progression into the next cell cycle phase (Fig 3B). Cells exposed to either HygB or PaT exhibited a rather small budding fraction (30%) that remained constant over time, indicating that the release from G1 is not affected but completion of the S-phase is prevented by HygB and as previously demonstrated [24] and confirmed here by PaT. Consistently, the *rad53-11* mutation increased toxicity of the drug (Fig 3A), which additionally agrees with the finding that HygB, as for PaT [24], is likely to provoke an S-phase arrest.

### HygB lesions are repaired by DSB repair

**Fig 4:**
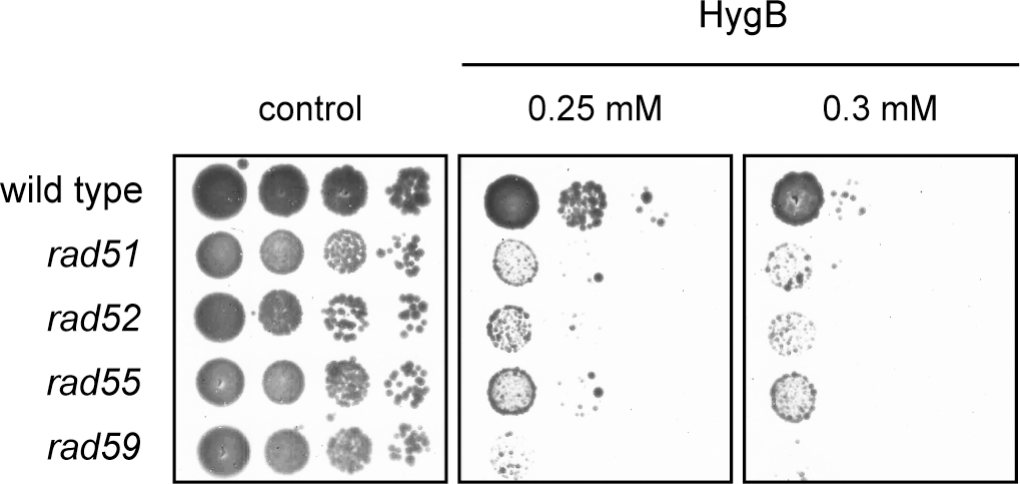
Relevance of DSB repair for hygromycin B action. *S*. *cerevisiae* strains defective in the *RAD52*-epistasis group (*rad52*, *rad55*, *rad57* and *rad59*) were tested by drop dilution assays against HygB.

Prolonged stalling of replication forks can cause fork collapse and formation of DSB intermediates which are recognized by the HR machinery but not by NHEJ [40–42], which agrees with the opposite responses obtained with HR (*rad52*) and NHEJ (*yku80*) deficient mutants, see Fig 1 and Klassen *et al*. [10]. As Rad52 inactivation is similarly detrimental for HygB survival (Fig 1), we suspected other members of the *RAD52* epistasis group to confer related phenotypes. Responses of *S*. *cerevisiae* cells defective in *RAD51*, *RAD52*, *RAD55* and *RAD59* to HygB was monitored by drop dilution assays (Fig 4). Inactivation of the eukaryotic RecA homolog Rad51 as well as the recombination mediators Rad52, Rad55 and Rad59 significantly aggravated HygB toxicity, supporting the conclusion that HygB may induce DSBs as a consequence of stalled replication forks, again a finding that is rather similar to PaT action [22].

A rather rapid cellular signal for DSB formation is the Mec1-dependent histone H2A phosphorylation on serine 129 [43]. As previously demonstrated for PaT [22], we also detected a prolonged phosphorylation level in HygB treated cells when compared to control cells (S3 Fig). The lasting phosphorylation status of histone H2A in response to HygB (and PaT) (120 min post G1-release) corroborates with the assumption that HygB causes secondary DSB formation as a consequence of replication fork stalling, although such effect appears to be more pronounced for PaT.

### The role of the BER in hygromycin B resistance

**Fig 5:**
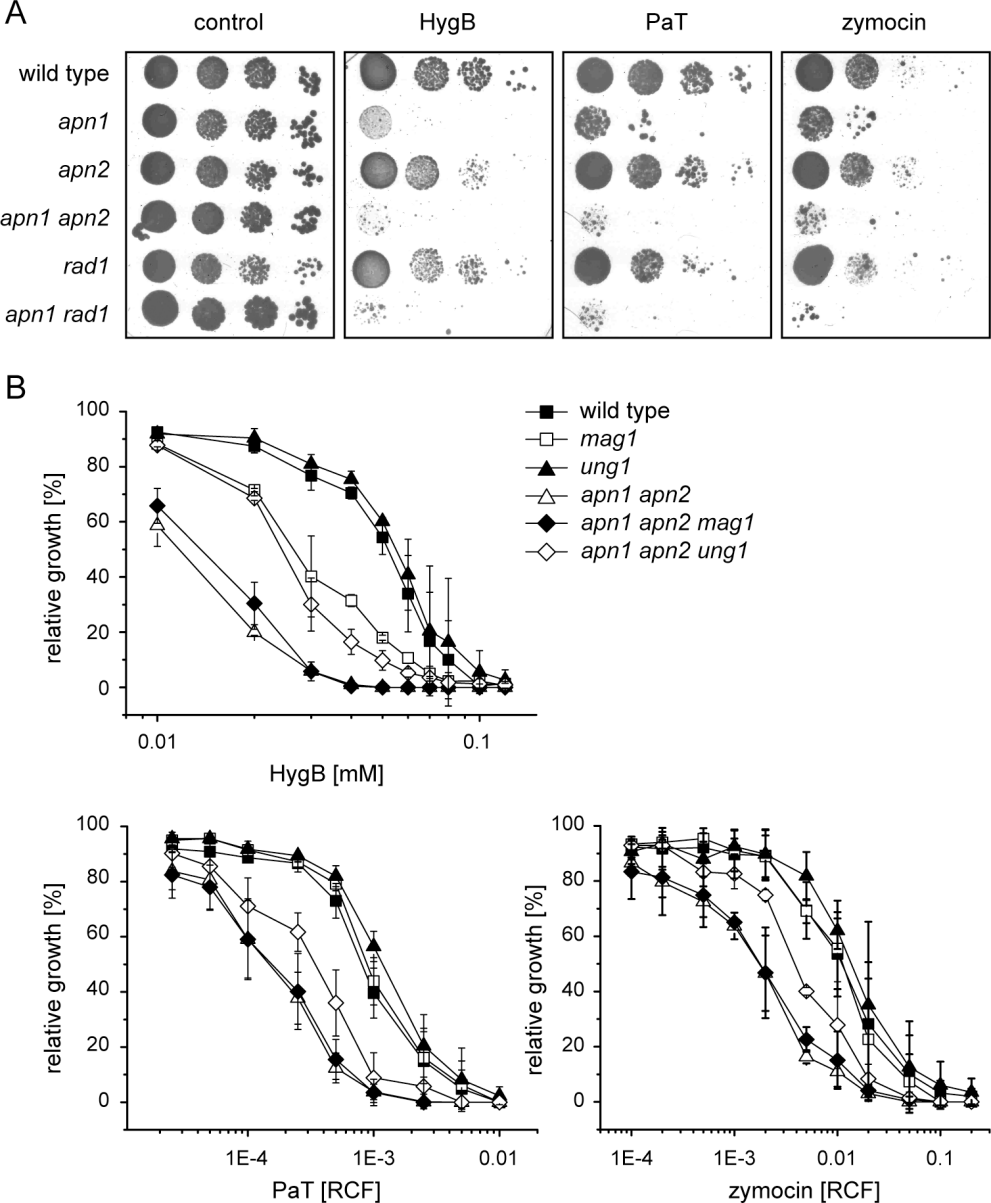
The role of BER for protection against hygromycin B. (A) Single or double mutants of *S*. *cerevisiae* defective in endonucleases processing AP-sites (*apn1*, *apn2* or *rad1*) were tested by drop dilution assays against HygB (2 mM), PaT (RCF 0.01) and zymocin (RCF 0.01), (B) cells defective in DNA glycosylases (*mag1*, *ung1*) and/or AP-site processing endonucleases (*apn1 apn2*) were tested by microtiter assays.

The HygB hypersensitivity of the BER mutant *apn1 apn2* defective in AP-site processing (Fig 1) suggests accumulation of abasic sites as for the ACNases [21]. AP-sites are highly toxic and mutagenic as they block DNA replication, induce misincorporations and provoke DSBs [44, 45]. The major pathway for processing such sites depends on Apn1 and Apn2 [46, 47], however, Rad1, an endonuclease instrumental in nucleotide excision repair (NER), can–as a backup activity—contribute to handling abasic sites as well [48, 49]. The role of Apn1/2 for HygB action was analyzed in drop dilution assays which were performed with the mutants (single and double); both ACNases PaT and zymocin were included for the purpose of comparison (Fig 5A). While loss of Apn1 clearly enhanced toxicity, inactivation of *APN2* or *RAD1* alone had no detectable effect, suggesting Apn1 to be the major AP endonuclease for providing protection, for PaT see also [21]. In the *apn1* background, however, mutations in *APN2* and *RAD1* displayed an additive sensitizing effect, suggesting that both endonucleases contribute to cell survival when Apn1 is disabled (Fig 5A).

AP-sites naturally arise either by spontaneous base hydrolysis or as intermediates during BER, in which specific DNA glycosylases recognize and remove aberrant bases [48]. Five DNA glycosylases with different substrate specificities are known in *S*. *cerevisiae*: Mag1 catalyzes the excision of alkylated purines from DNA [50], Ogg1 removes 8-oxoguanine [51, 52], while a variety of defective (predominantly oxidized) pyrimidines are repaired by Ntg1 and Ntg2 [53]; excision of uracil involves the DNA glycosylase Ung1 [54]. For checking whether DNA base damage is causative for AP-site formation, cells defective in DNA glycosylase activity and/or AP-site repair were tested against HygB (Fig 5B and S4 Fig). The sensitivity profiles of the mutants were compared to those obtained with zymocin and PaT (Fig 5B and 5C) [21]. Inactivation of the DNA glycosylases involved in the repair of oxidative damages (*ogg1*, *ntg1*, *ntg2* and *ntg1 ntg2*) had no detectable effect with respect to HygB toxicity (S4 Fig), again as for zymocin and PaT [21]. In contrast to both ACNase toxins, deletion of *MAG1* enhanced HygB sensitivity (Fig 5B). This result indicates a role of Mag1 in the repair of endogenous alkylation damage caused by HygB, which agrees with findings demonstrating genome wide DNA hypermethylation upon HygB treatment in *Nicotiana tabacum* [55]. The increased sensitivity of *apn1 apn2* compared to *mag1* suggests that unprocessed AP lesions are more toxic than alkylated bases. However, disabling Mag1 did not significantly influence the *apn1 apn2* associated sensitivity, contrary to MMS [56]. If HygB induced AP-site formation was exclusively via excision of alkylated bases by Mag1, a strong rescue of HygB sensitivity would be expected in the *apn1 apn2 mag1* background. The absence of such phenotypic rescue may point to other mechanisms for AP-site formation in HygB treated cells. Indeed, similar to PaT [21], deletion of *UNG1* partially suppressed the HygB and zymocin sensitivity in the *apn1 apn2* mutant (Fig 5B) adverting to uridine excision by Ung1 as the likely source of AP-site formation. In the DNA, uridine may occur either by cytosine deamination or, more likely, by misincorporation of dUMP [48]. The latter may be—due to translational inhibition—a consequence of reduced Dut1 (dUTPase) levels. Since Dut1 catalyzes the conversion of dUTP to dUMP, the precursor for dTTP-synthesis, HygB/ACNase treatment may interfere with Dut1 formation or activity, thereby—by blocking dTTP-synthesis—promoting incorporation of dUTP into DNA [57]. Since replicative DNA polymerases do not distinguish uridine from thymidine [58], replication across uridine is not obstructed, which agrees with the observed slightly enhanced tolerance of the *ung1* mutant (Fig 5B). Abasic DNA lesions are far more toxic than incorporated uracil bases [59, 60] reasoning the reduced toxicity of the translational inhibitors when *UNG1* is deleted in the *apn1 apn2* background (Fig 5B) and [21]. These genetic lines of evidence suggest that in response to HygB treatment, Ung1 creates products that become toxic in the absence of AP-site repair.

### Post replication repair contributes to hygromycin B resistance

Post replication repair (PRR) is critical for genome stability, as it provides a mechanism to bypass unrepaired DNA lesions that would otherwise hamper replication fork progression [61, 62]. The PRR pathway consists of three branches alternatively followed depending on the degree of modification of the proliferating cell nuclear antigen (PCNA) [63, 64]. Mono-ubiquitination of PCNA by Rad6-Rad18 stimulates translesion synthesis (mutagenic or error-free) via the DNA polymerases ζ (Rev3/Rev7) or η (Rad30) [65–67], whereas multi-ubiquitination by Rad5-Ubc13-Mms2 promotes error-free damage avoidance via template switching [68, 69]. In its sumoylated form, PCNA serves to channel lesion repair into the PRR pathway, recruiting helicase Srs2 to the replication forks, where it blocks objectionable recombination events by disrupting Rad51-filaments [70, 71].

**Fig 6:**
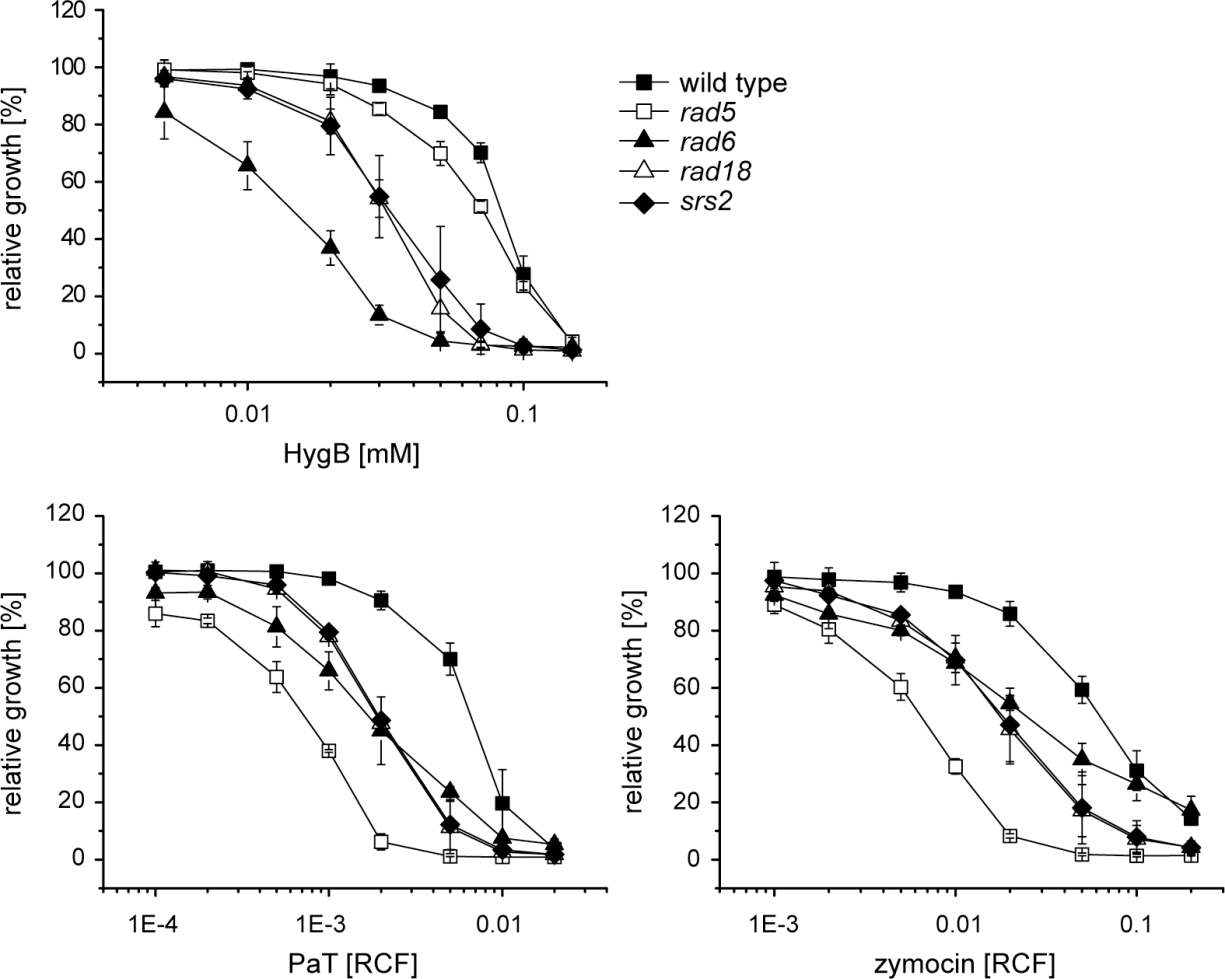
Inactivation of PRR genes enhances toxicity of translational inhibitors. The sensitivity of *S*. *cerevisiae* strains defective in Rad5, Rad6, Rad18 or Srs2 were checked with HygB, PaT and zymocin in microtiter assays.

As PRR was previously shown to be important for handling stalled replication forks induced by the ACNase PaT [21], we analyzed its relevance for HygB and zymocin action (Fig 6). Mutations in *RAD6*, *RAD18*, *RAD5* and *SRS2* similarly, as for PaT, increased sensitivity to zymocin, implicating that PRR is equally vital for zymocin cell survival, possibly by preventing replication fork collapse into DSBs [21]. Compared to the ACNase toxins, there is a different strategy to tolerate HygB. Sensitivity of *rad6* is increased with respect to *rad18*, while *rad5* appears to be minor affected (Fig 6). As Rad6 is required for a variety of other cellular functions than DNA repair [72], presumably a different Rad6-involving mechanism, besides PRR, contributes to HygB survival. Interestingly, inactivation of the anti-recombinase Srs2 increased sensitivity to all of the three inhibitors, see Fig 6 and [21]. Srs2 is proposed to function as a switch for PRR and HR [73]; when PRR is disabled (*rad6*), Srs2 deters HR from repair of collapsed replication forks, thereby aggravating ACNase and HygB toxicity (Fig 6). Simultaneous deletion of *SRS2*, however, nullifies the block to recombination (when HR is active), which consequently resulted in a suppressed *rad6* phenotype towards PaT and UV light, as previously demonstrated [21]. Thus, a functional PRR, in addition to HR and BER, is pivotal for genome stability and cell viability, likely by suppressing fork collapse and DSB formation in response to ACNase or HygB treatment.

### Polymerase ζ contributes to mutagenesis and toxicity

**Fig 7:**
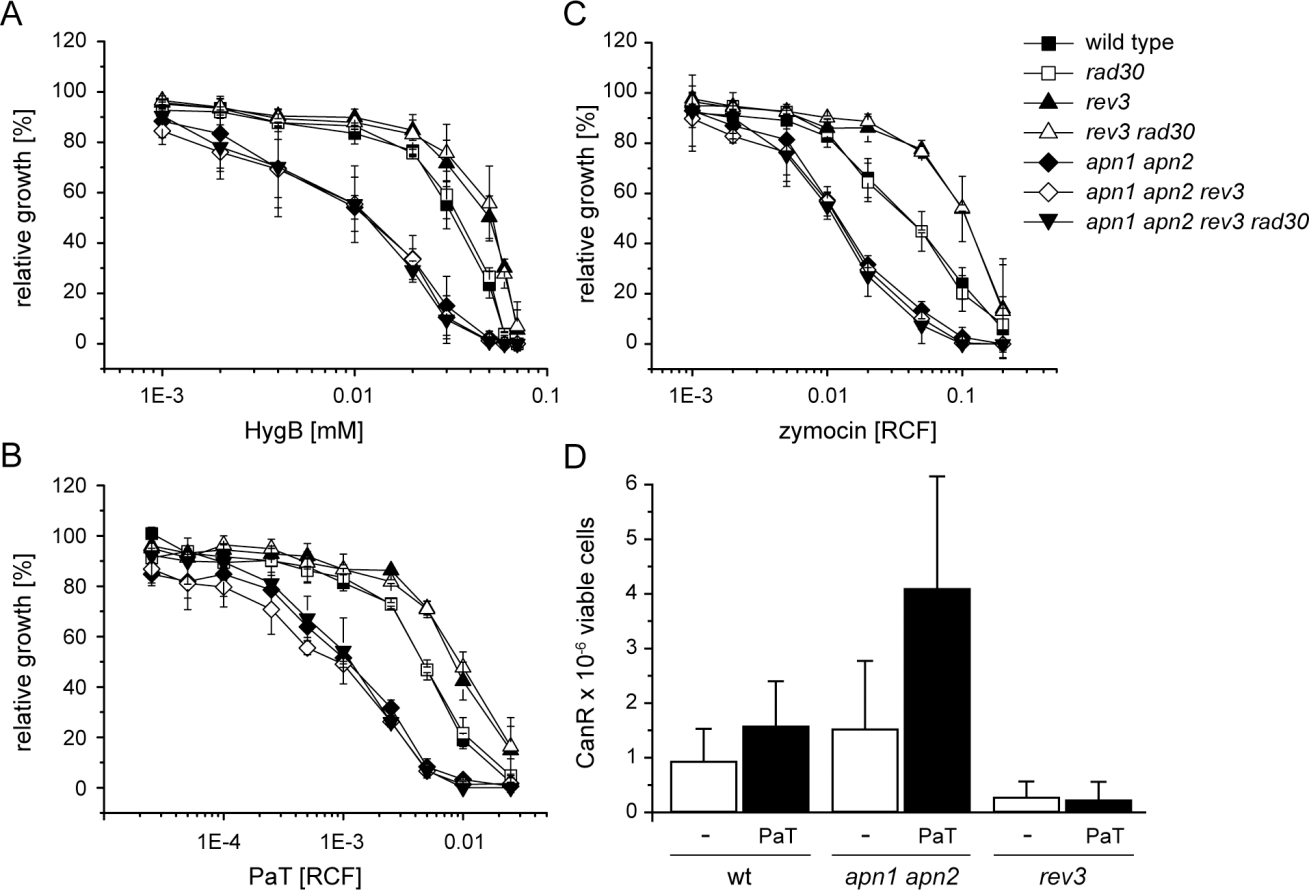
Polymerase ζ contributes to toxicity of translational inhibitors. The sensitivity of *S*. *cerevisiae* strains defective in error-prone polymerase ζ (*rev3*), error-free polymerase η (*rad30*) and/or AP endonucleases (*apn1 apn2*) was tested against HygB (A), zymocin (B) and PaT (C) by microtiter assays. (D) Mutation rates at the *CAN1* locus were exemplarily determined for PaT in wild type, *apn1 apn2* and *rev3* cells, when exposed to PaT. Standard deviations are depicted as error bars.

The role of TLS, which is again controlled by the PRR pathway [74], was studied by recording HygB and ACNase sensitivity of *rev3* and *rad30* mutants, defective in the error-prone polymerase (Pol) ζ or the error-free Pol η, respectively (Fig 7A–7C). While deletion of *RAD30* had no effect, inactivation of Pol ζ by deleting its catalytic subunit (*REV3*) increased resistance of the wild type as well as the *rad30* genetic background, which suggests Pol ζ to be responsible for the mutagenic effects observed with all of the three inhibitors, see Fig 2 and Klassen and Meinhardt [23]. To check such assumption, mutation rates were determined at the *CAN1* locus in PaT treated and untreated cells lacking *REV3* and compared to those in the likewise treated wild type cells (Fig 7D). As anticipated from previous findings, PaT treatment increased mutation frequencies in the wild type [23] but, most strikingly, loss of *REV3* entirely prevented mutagenicity, evidencing that Pol ζ is responsible for PaT’s mutagenic effects. Consistently, *rev3* mutations increased resistance, not only to PaT but also to zymocin and HygB (Fig 7A–7C), thereby indicating that mutations brought about by Pol ζ contribute to the toxic potential of the three translational inhibitors.

Unlike Pol η, which is not relevant for toxin action, as judged from the wild type like toxin response of the *rad30* mutant to all of the three agents (Fig 7A–7C), Pol ζ can mediate replication past a large variety of lesions, including AP-sites [74, 75]. To determine, whether the mutagenic effect results from error-prone TLS across AP-sites, the influence of AP-site repair on PaT-induced mutations was checked by monitoring the mutation rates in a strain lacking Apn1 and Apn2 (Fig 7D). Interestingly, loss of AP endonucleases significantly increased the number of random as well as toxin induced mutations, suggesting that in the absence of AP-site repair, bypass of toxin-mediated lesions occurs through Pol ζ, similar to the Polζ-mediated bypass of MMS-induced DNA damages [46]. Elimination of the error-prone (*rev3*) or the complete TLS pathway (*rev3 rad30*) in *apn1 apn2* did no longer reveal a protective effect to any of the three inhibitors (Fig 7A–7C), thereby disclosing two compensating effects of error-prone TLS: (i) DNA damage manifestation by mutation induction at AP-sites and (ii) conferring the capability to continue replication in the presence of fork stalling AP-sites. Evidently, unrepaired AP-sites and the thereby imposed replication block are extremely toxic. It should, however, taken into consideration that the observed effect of Pol ζ may be due to its backup activity in DNA replication seen under replication fork stalling conditions in HU-treated cells [76].

Hygromycin B exerts its effect on translation by interfering with the ribosomal translocation and mRNA-decoding process [7], whereas both ACNase toxins target translation at the step of tRNA supply [9, 10]. Despite different modes of action, sensitivity analyses of DNA repair mutants revealed a similar destabilizing effect on genome integrity. Based on these genetic evidences, we suggest that translational hindrance by HygB elicits loss of DNA integrity accompanied by an increase in mutagenesis, which presumably involves replication fork stalling and collapse into DSBs as for PaT [10, 21–25]. Consistently, an S-phase arrest is induced upon activation of the DNA damage checkpoint. The protective effects of DNA repair mechanisms, such as BER and HR, as well as PRR as a bypass mechanism, underline the importance of handling stalled forks upon HygB exposure. It remains to be determined, however, whether the mutagenic effect of Pol ζ is attributed via error-prone TLS (across AP-sites) or is simply due to the backup function in DNA replication.

Our results with neomycin, geneticin and cycloheximide (S1 Fig) may suggest that DNA damaging effects are not limited to the ACNases and HygB. Hence, translational stress probably impacts genome integrity in general. Since the DNA repair mutants do not react uniformly to the drugs (Fig 1 and S1 Fig), additional analyses of the way they impact genome stability are necessary. Only rather recently, we demonstrated that the ACNase PaT induced DNA damage as a consequence of ribonucleotide reductase (RNR) depletion [25]. Paromomycin, known to cause amino acid misincorporations and translational frameshifting by disturbing the ribosomal decoding center [77], was seen to reduce RNR levels as well; it was assumed [26] that the exerted translational stress activates protein stress response pathways which ultimately leads to RNR degradation. Thus, two translational inhibitors (paromomycin and PaT ACNase) targeting different aspects of the translation process directly interfere with replication fork progression by reducing dNTP supply. Our results suggest that this might be a more generalized phenomenon occurring with additional translational inhibitors as well and indicate that inhibition of dTTP formation may also contribute to DNA damage arising under translational stress.

The genetic analyses outlined in this contribution furnish evidence that translational antibiotics such as HygB–against expectation–can be mutagenic. These novel findings may have a direct impact on the use of such drugs for therapeutic purposes as generation of resistance phenotypes by concomitantly elicited mutations can not *a priori* be ruled out.

## Supporting Information

S1 Fig Relevance of DNA repair mechanisms for the protection against ribosomal inhibitors. Microtiter assays were performed with *S*. *cerevisiae* strains deficient in homologous recombination (*rad52*), non-homologous end-joining (*yku80*), base excision repair (*apn1 apn2*) or post replication repair (*rad18*). Relative growth was determined photometrically at 620 nm and corresponds to the OD value of strains cultivated in medium without antibiotics. Standard deviations of three biological replicates (with two technical replicates for each) are represented by the error bars.
(TIF)

S2 Fig Sensitivity of DNA repair mutants to UV irradiation, MMS and hydroxyurea (HU) treatment. *S*. *cerevisiae* strains deficient in homologous recombination (*rad52*), non-homologous end-joining (*yku80*), base excision repair (*apn1 apn2*) or post replication repair (*rad18*) were spotted as serial dilutions onto YPD plates and exposed to 180 J/m^2^ UV irradiation or spotted on medium containing 0.4% MMS or 100 mM HU.
(TIF)

S3 Fig Histone H2A phosphorylation in cells exposed to HygB or PaT. Alpha-factor arrested *S*. *cerevisiae* KY117 cells were released to toxin-free or medium supplemented either with HygB, MMS or PaT. Samples were taken at the α-factor mediated G1-arrest, after 30 min of MMS exposure and at indicated intervals post G1-release. The phosphorylation status of histone H2A was monitored by Western blot analysis applying a polyclonal antibody raised against the serine 129 phosphorylated protein. The coomassie-stained protein fraction is shown as the loading control. DSBs routinely occur during replication, and thus accumulate during the S-phase of the cell cycle. Consistently, in the G1-arrested cells only a small amount of phosphorylated histone H2A could be detected. Mock cells released to the S-phase (YPD) exhibited—as to be expected—a strong increase of the phosphorylation level that rapidly decreased after 60 min post G1-release upon progression into the next phase of the cell cycle. HygB exposed cells (as for the mutagenic MMS and PaT) increased the phosphorylation level of histone H2A. The phosphorylation status is maintained for a longer period of time than for mock cells; an increase is seen even after 120 min post G1-arrest followed by a slight reduction after 180 min. Agreeing with a previous study [22] PaT treated cells displayed a constantly high phosphorylation level during the entire monitored period.
(TIF)

S4 Fig Role of DNA glycosylases involved in the repair of oxidative DNA damage. *S*. *cerevisiae* strains defective in DNA glycosylases (Ogg1, Ntg1 and/or Ntg2) and/or AP-site processing endonucleases (Apn1 and Rad1) were tested against hygromycin B (HygB), PaT and zymocin by microtiter assays. A relative concentration factor of 1 (RCF 1) equals the toxin concentration in the supernatant of a stationary phase culture of *P*. *acaciae* or *K*. *lactis*.
(TIF)
