# Supplementary figures and images for: DNA Damage Responses Are Induced by tRNA Anticodon Nucleases and Hygromycin B
Source: PLoS One. 2016 Jul 29;11(7):e0157611. doi: 10.1371/journal.pone.0157611 (PMC4966947; doi:10.1371/journal.pone.0157611)

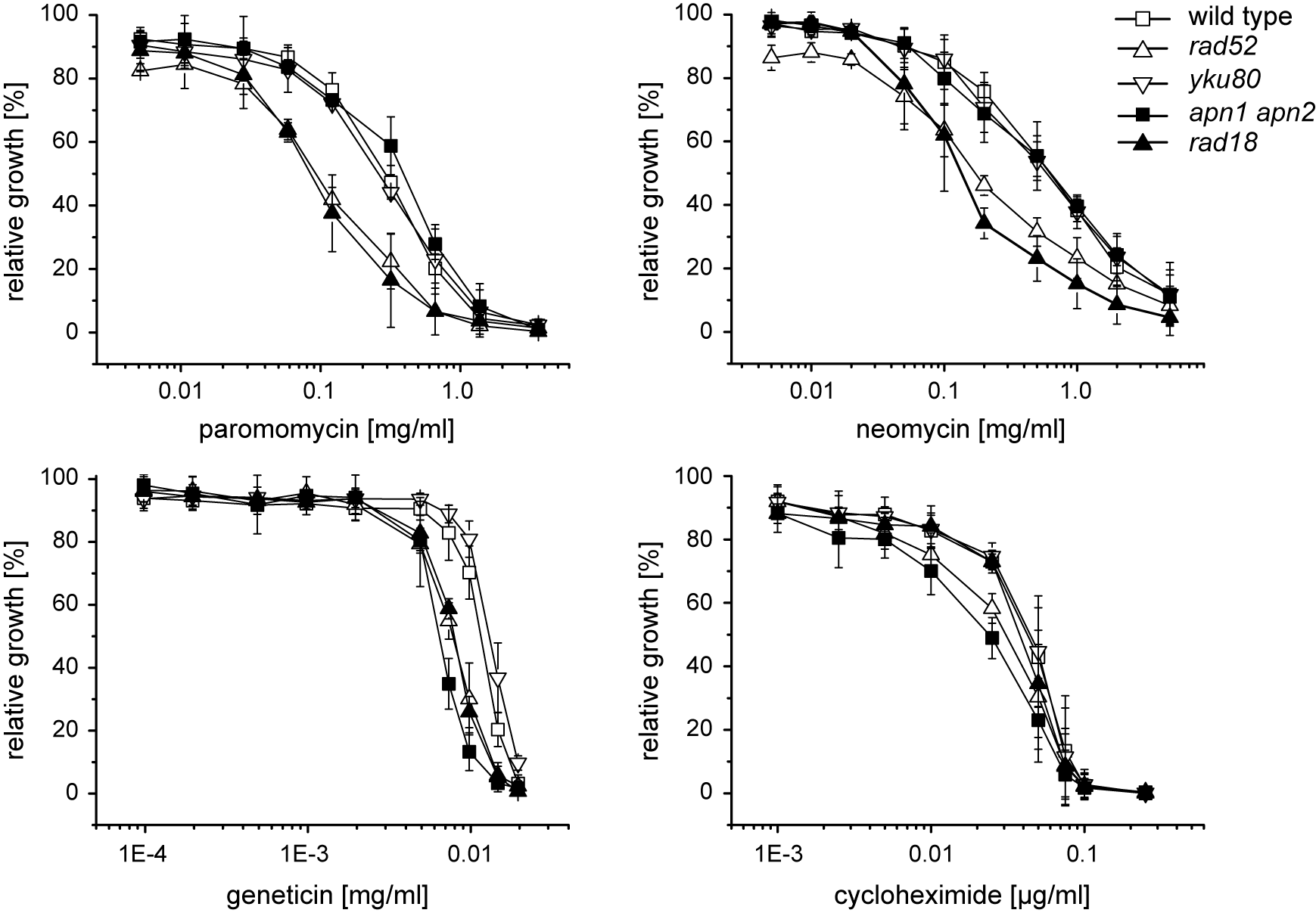

Supplement: S1 Fig — Microtiter assays were performed with S. cerevisiae strains deficient in homologous recombination (rad52), non-homologous end-joining (yku80), base excision repair (apn1 apn2) or post replication repair (rad18). Relative growth was determined photometrically at 620 nm and corresponds to the OD value of strains cultivated in medium without antibiotics. Standard deviations of three biological replicates (with two technical replicates for each) are represented by the error bars. (TIF) [file pone.0157611.s001.tif]

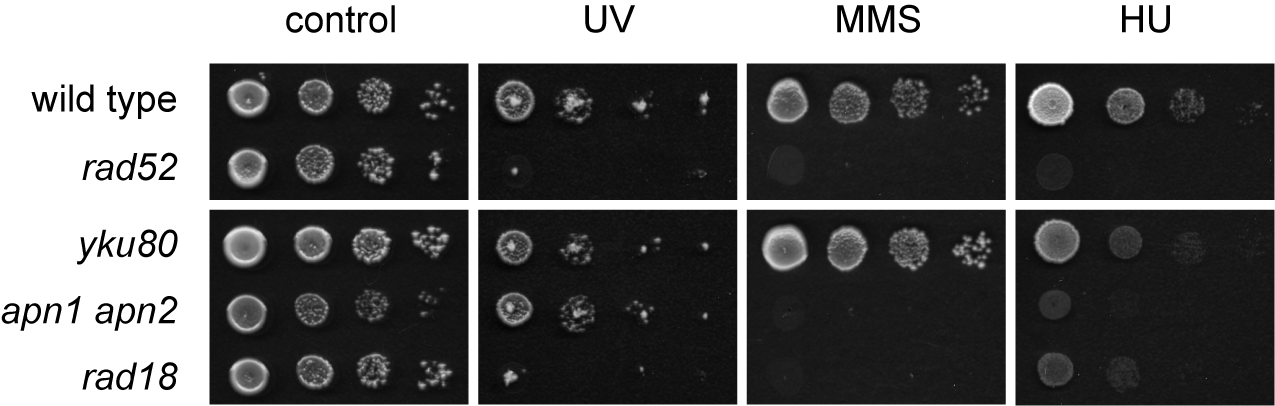

Supplement: S2 Fig — S. cerevisiae strains deficient in homologous recombination (rad52), non-homologous end-joining (yku80), base excision repair (apn1 apn2) or post replication repair (rad18) were spotted as serial dilutions onto YPD plates and exposed to 180 J/m2 UV irradiation or spotted on medium containing 0.4% MMS or 100 mM HU. (TIF) [file pone.0157611.s002.tif]

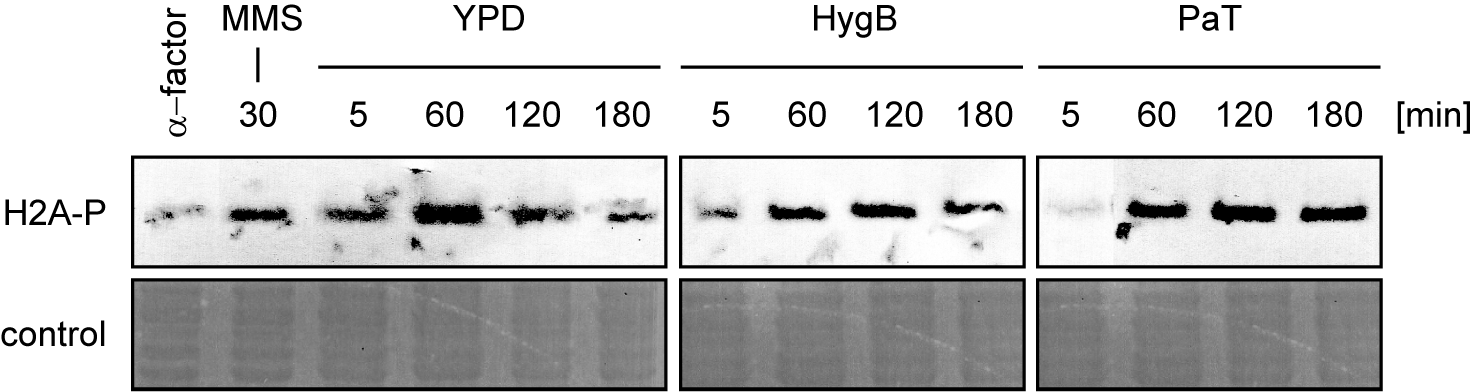

Supplement: S3 Fig — Alpha-factor arrested S. cerevisiae KY117 cells were released to toxin-free or medium supplemented either with HygB, MMS or PaT. Samples were taken at the α-factor mediated G1-arrest, after 30 min of MMS exposure and at indicated intervals post G1-release. The phosphorylation status of histone H2A was monitored by Western blot analysis applying a polyclonal antibody raised against the serine 129 phosphorylated protein. The coomassie-stained protein fraction is shown as the loading control. DSBs routinely occur during replication, and thus accumulate during the S-phase of the cell cycle. Consistently, in the G1-arrested cells only a small amount of phosphorylated histone H2A could be detected. Mock cells released to the S-phase (YPD) exhibited—as to be expected—a strong increase of the phosphorylation level that rapidly decreased after 60 min post G1-release upon progression into the next phase of the cell cycle. HygB exposed cells (as for the mutagenic MMS and PaT) increased the phosphorylation level of histone H2A. The phosphorylation status is maintained for a longer period of time than for mock cells; an increase is seen even after 120 min post G1-arrest followed by a slight reduction after 180 min. Agreeing with a previous study [22] PaT treated cells displayed a constantly high phosphorylation level during the entire monitored period. (TIF) [file pone.0157611.s003.tif]

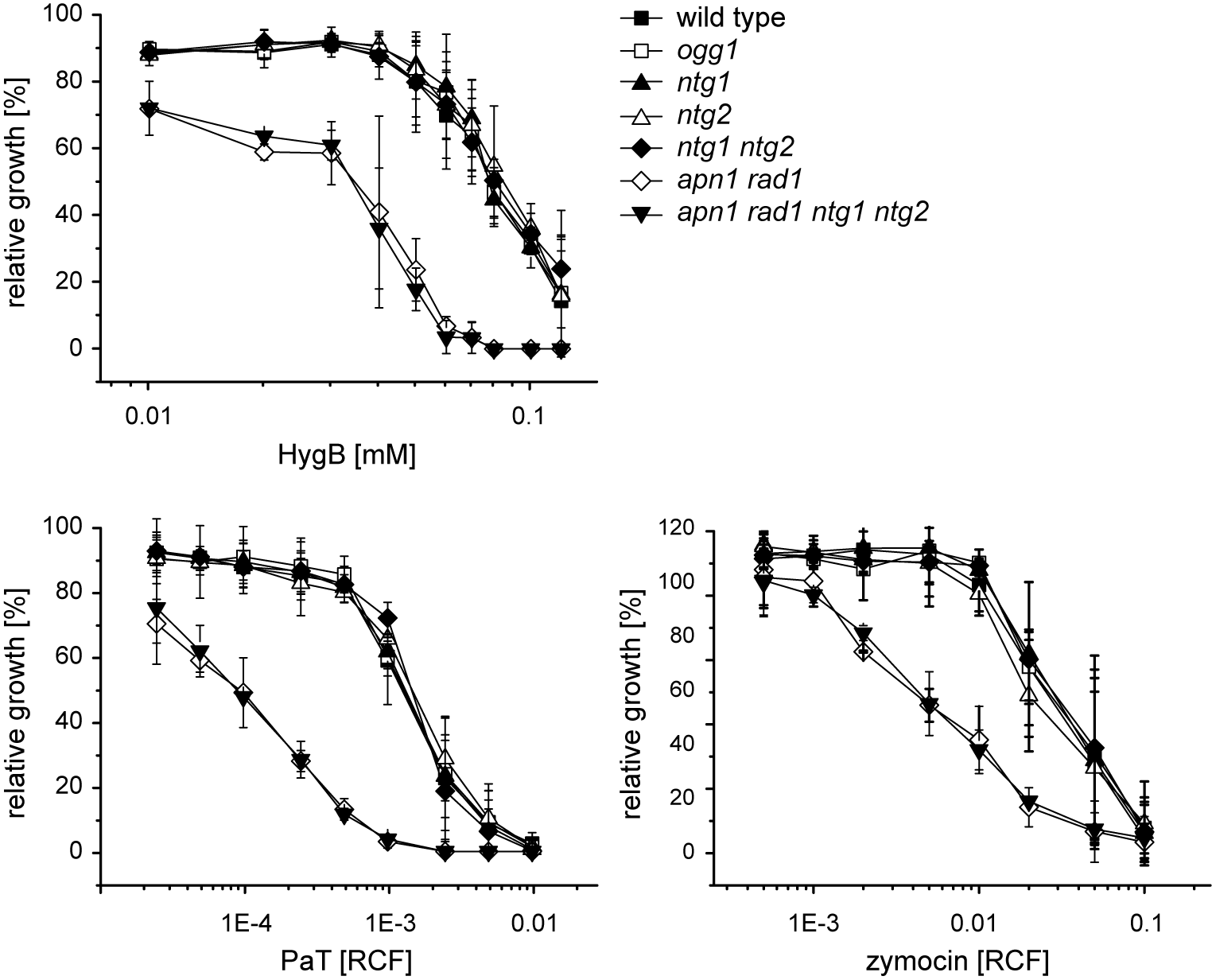

Supplement: S4 Fig — S. cerevisiae strains defective in DNA glycosylases (Ogg1, Ntg1 and/or Ntg2) and/or AP-site processing endonucleases (Apn1 and Rad1) were tested against hygromycin B (HygB), PaT and zymocin by microtiter assays. A relative concentration factor of 1 (RCF 1) equals the toxin concentration in the supernatant of a stationary phase culture of P. acaciae or K. lactis. (TIF) [file pone.0157611.s004.tif]
